# Challenges and barriers of using large language models (LLM) such as ChatGPT for diagnostic medicine with a focus on digital pathology – a recent scoping review

**DOI:** 10.1186/s13000-024-01464-7

**Published:** 2024-02-27

**Authors:** Ehsan Ullah, Anil Parwani, Mirza Mansoor Baig, Rajendra Singh

**Affiliations:** 1 Anatomical Pathology, Department of Pathology and Laboratory Medicine, Te Toka Tumai Auckland, Te Whatu Ora (Health New Zealand), Auckland, New Zealand; 2 Department of Pathology, Wexner Medical Center, The Ohio State University, Columbus, OH USA; 3 Health Intelligence, Orion Health, Auckland, New Zealand; 4 Director of Dermatopathology and Digital Pathology, Summit Health, Woodland Park, NJ USA

**Keywords:** Large learning models, LLMs, ChatGPT, Challenges and barriers of using LLMs, AI, ML, Diagnostic medicine, Pathology, Digital pathology

## Abstract

**Background:**

The integration of large language models (LLMs) like ChatGPT in diagnostic medicine, with a focus on digital pathology, has garnered significant attention. However, understanding the challenges and barriers associated with the use of LLMs in this context is crucial for their successful implementation.

**Methods:**

A scoping review was conducted to explore the challenges and barriers of using LLMs, in diagnostic medicine with a focus on digital pathology. A comprehensive search was conducted using electronic databases, including PubMed and Google Scholar, for relevant articles published within the past four years. The selected articles were critically analyzed to identify and summarize the challenges and barriers reported in the literature.

**Results:**

The scoping review identified several challenges and barriers associated with the use of LLMs in diagnostic medicine. These included limitations in contextual understanding and interpretability, biases in training data, ethical considerations, impact on healthcare professionals, and regulatory concerns. Contextual understanding and interpretability challenges arise due to the lack of true understanding of medical concepts and lack of these models being explicitly trained on medical records selected by trained professionals, and the black-box nature of LLMs. Biases in training data pose a risk of perpetuating disparities and inaccuracies in diagnoses. Ethical considerations include patient privacy, data security, and responsible AI use. The integration of LLMs may impact healthcare professionals’ autonomy and decision-making abilities. Regulatory concerns surround the need for guidelines and frameworks to ensure safe and ethical implementation.

**Conclusion:**

The scoping review highlights the challenges and barriers of using LLMs in diagnostic medicine with a focus on digital pathology. Understanding these challenges is essential for addressing the limitations and developing strategies to overcome barriers. It is critical for health professionals to be involved in the selection of data and fine tuning of the models. Further research, validation, and collaboration between AI developers, healthcare professionals, and regulatory bodies are necessary to ensure the responsible and effective integration of LLMs in diagnostic medicine.

## Introduction

ChatGPT (Chatbot Generative Pre-Trained Transformer) is one of the latest and most powerful Large Language Models (LLM). Its disruptive capabilities have taken the globe by storm, opening a new era of possibilities and challenges in every field including healthcare. Despite ChatGPT not being trained on medical data, medical professionals and researchers have trialed to analyze and interpret medical data demonstrating multiple utilities of this chatbot in research and practice settings [1]. European Federation of Clinical Chemistry and Laboratory Medicine (EFLM) Working Group on Artificial Intelligence (WG-AI) studied the capability of ChatGPT [2] and reported that in its current form, despite being not specifically trained on laboratory medicine data, it could still detect abnormal test results according to reference intervals on a test-to-test basis. However, it could not interpret an overall diagnostic picture. A study [2] reported that ChatGPT could recognize hypothetically created set of eight scenario-based microbiology questions and responded accurately, providing appropriate antimicrobial spectra and regimens for the diagnosis. When it came to highlighting the implications of clinical response, predict the correct length of antimicrobial therapy and recognize complex management considerations (e.g. blood culture contamination) its responses however had some important limitations [1, 2]. Initial development in the area of clinical decision support systems, smart systems and AL/ML-based advancements in the healthcare have initiated a good platform for LLM to be integrated and utilized efficiently in the healthcare space [3–6].

Future iterations of generative AI tools such as ChatGPT, with pre-training on specific laboratory medicine data, could assist laboratory medicine professionals by allowing quick analysis of large volumes of test results and other laboratory data, identification of patterns, making correlations between test results, clinical notes to generate interpretations (decision support functions); recognising aberrant and outlier test results and pick up other quality control issues; enhancing and optimizing laboratory workflows by identifying process inefficiencies; and serving as an educational and research tool.

Similarly, ChatGPT could be coupled with computer vision models to enhance utility in computational pathology. ChatCAD [6] is one such model that combines ChatGPT with a disease classifier and lesion detector to diagnose radiology images, promises remarkable opportunities for development of interactive chatbots that could make computer assisted diagnosis into a reality. SkinGPT [7] combines ChatGPT and a visual model trained on skin images dataset. It allows users to upload their own skin photos for diagnosis. It determines the macroscopic features of skin lesions, analyses those features, and provides diagnosis and treatment recommendations. Segment anything model (SAM) shows good hope to serve as a benchmark model of computer vision for medical images and presents remarkable results when tested on a large public dataset of multimodality radiological images from various organs representing assorted pathologies.

Despite above mentioned possibilities, there are several challenges to fully combine ChatGPT and other LLMs with suitable computer vision models to develop interactive chatbot that would allow users to diagnose a condition on histological images such as whole slide images. Some of these barriers include lack of sufficient histology image datasets available publicly to validate such models, quality control issues related to tissue processes and staining; and complexity of data (hematoxylin and eosin-stained images, to immunohistochemistry markers and other molecular testing to electron microscopy images, often requiring correlation of microscopic features). These issues make it difficult for models to learn accurate representations of images. Moreover, there are certain concerns of using LLMs to combine it with the visual images to develop CAD tools. Those issues including but not limited to the lack of transparency of the AI algorithms used by LLMs to interpret data; chances of inadvertently perpetuating biases based on the type of datasets used to train the model thus potential to discriminate against certain patient populations; ethical concerns related to privacy and informed consent; legal and regulatory considerations [1, 2, 6, 7].

Despite these challenges and issues, revolution of current healthcare processes including those of laboratory medicine including digital and computational pathology are inevitable with future generations of generative AI tools trained on medical ground truth data and combined with specialized and validated visual transformers [3, 7, 8].

## Opportunities, challenges, and barriers of using LLM for diagnostic medicine

Large language models have the potential to revolutionize diagnostic medicine, including digital pathology. However, it’s important to consider both the pros and cons of using these models in such applications. Here are some key points to consider [9, 10]:

### Opportunities

1. Knowledge Access: Large language models have access to vast amounts of medical literature, research papers, clinical guidelines, and other relevant sources. They can quickly retrieve and synthesize information, enabling healthcare providers to access a wealth of knowledge to aid in diagnosis and treatment decisions.
2. Diagnostic Support: Language models can assist pathologists and other healthcare professionals in analyzing digital pathology images. They can help identify patterns, highlight potential abnormalities, and provide additional insights based on existing medical knowledge. This can enhance accuracy, efficiency, and consistency in diagnosis.
3. Accessibility and Scalability: Language models can be accessed through digital platforms, making them easily available to healthcare professionals regardless of their geographic location. This accessibility allows for widespread adoption and scalability, potentially benefiting a larger number of patients.
4. Continuous Learning: Language models can be continuously trained and updated with the latest medical information, ensuring that they stay current and incorporate the most recent advancements in the field of digital pathology. This adaptability enables the model to improve over time and provide more accurate and reliable diagnostic support.

### Challenges

1. Lack of Contextual Understanding: While current language models have access to vast amounts of medical information, they may lack contextual understanding of specific patient cases or the nuances of individual diseases as they have not been specifically trained for medical tasks. They rely solely on the data and information provided to them, which can limit their ability to make accurate diagnoses in complex or unique cases.
2. Limited Interpretation: Language models primarily provide text-based outputs and may not have the ability to interpret visual information, such as complex digital pathology images. While they can provide insights based on existing knowledge, they may not fully grasp the intricacies and visual cues that pathologists rely on for diagnosis.
3. Ethical and Legal Concerns: Using language models for diagnostic medicine raises ethical and legal concerns. Patient privacy, data security, liability, and accountability are important considerations when deploying these models in clinical settings. Ensuring proper consent, protecting patient information, and mitigating potential biases in the models are critical aspects that need to be addressed.
4. Overreliance and Dependency: There is a risk of overreliance on language models, potentially leading to reduced critical thinking or independent decision-making by healthcare professionals. It is crucial to view these models as tools to augment human expertise rather than replace it entirely.
5. Bias and Error Propagation: Language models are trained on vast amounts of data, which can introduce biases present in the data itself. If the training data is biased or contains errors, the model may inadvertently propagate these biases or errors in its outputs. Careful attention must be given to training data selection and ongoing bias detection and mitigation efforts.

**Fig. 1:**
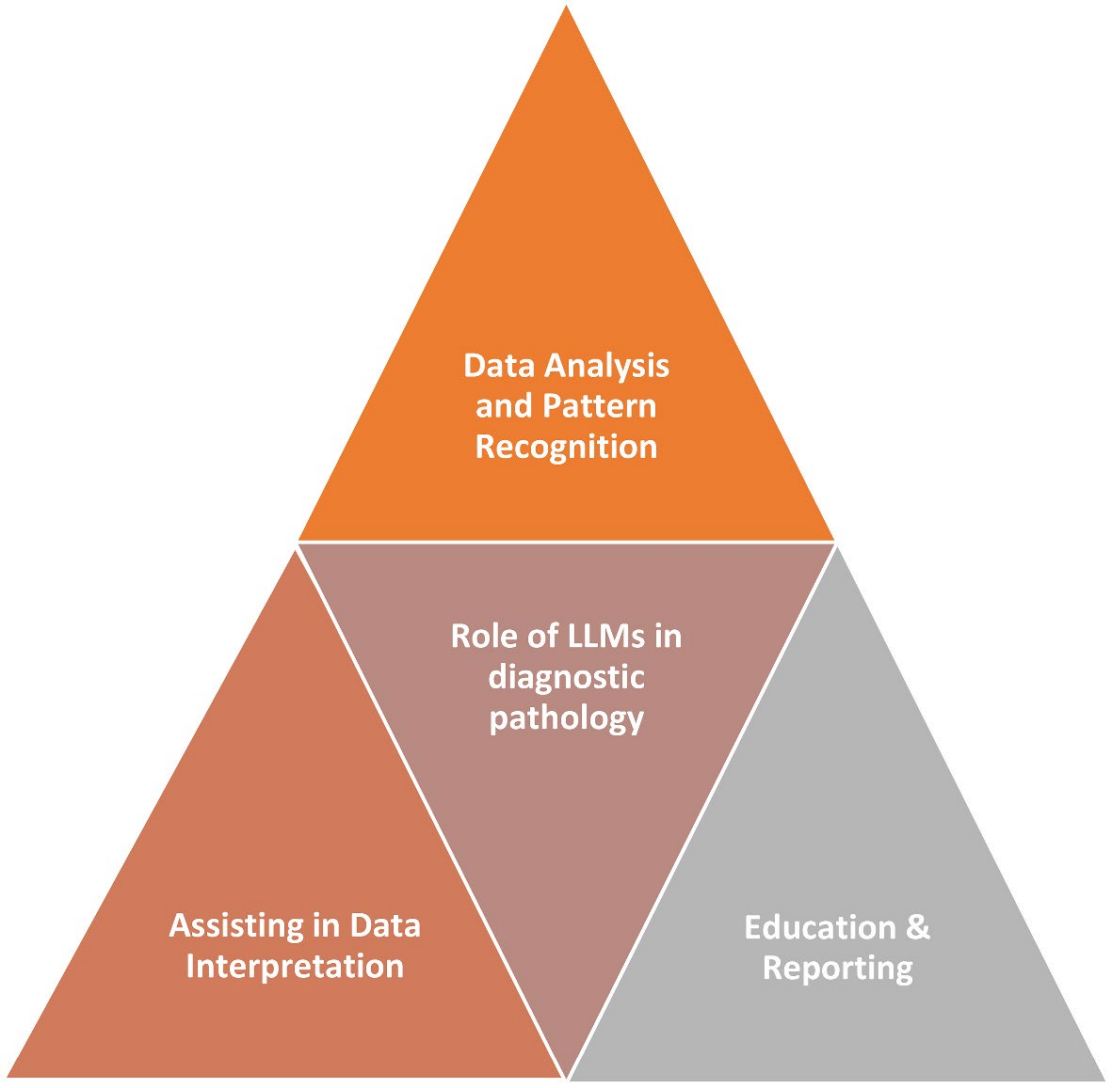
A schematic diagram illustrating the role of LLMs in diagnostic pathology

In summary, while LLMs offer several potential benefits for diagnostic medicine, particularly in digital pathology, it is important to be mindful of their limitations and potential risks. Careful implementation, validation, and ongoing monitoring are essential to ensure that these models are used as valuable tools in (Fig. 1) conjunction with human expertise, rather than as standalone diagnostic systems [10–12].

## Methodology

### Article search and selection criteria

In recent years, the emergence of LLMs, has sparked interest in their potential applications in diagnostic medicine. These models, powered by deep learning algorithms, have demonstrated the ability to generate human-like text and provide conversational responses. The use of LLM in diagnostic medicine holds promise for improving clinical decision-making, enhancing patient engagement, and optimizing healthcare delivery. However, it is important to critically examine the strengths, limitations, and ethical considerations associated with the use of LLM in this context.

The purpose of this critical review is to evaluate the current state of knowledge regarding the application of LLM in diagnostic medicine. Specifically, this review will assess the potential benefits, address the challenges, and explore the ethical implications of integrating LLM into clinical practice.

To conduct this review, a comprehensive search was conducted using all major electronic databases, including PubMed, Google Scholar, IEEE, Springer Link and relevant medical informatics journals. The query and keywords used were “ChatGPT,” “diagnostic medicine,” “clinical decision support,” “medical diagnosis,” ‘LLM in digital pathology’, ‘use of AI in digital pathology’ and “artificial intelligence” were used to identify relevant articles published within the past five years. The selected articles were critically analysed to extract key findings and insights.

The eligibility criteria for inclusion in the review are:

1. Original articles only published as a journal article.
2. Papers published or reported between 2020 and 2023 (inclusive).
3. The use of LLM in digital pathology and diagnostic medicine was the primary subject of this study.
4. Written and published in English.

We excluded articles that were not considered original research, such as letters to editors, comments, or reviews.

### Results

Initially, 189 studies were identified through database searching. A total of 144 records did not meet our inclusion criteria based on the initial screening, and therefore, 45 studies were included for checking against the eligibility. Full-text papers were retrieved and reviewed by two authors for completeness and quality. After the exclusion criteria applied, seven articles were deemed suitable for the final review by all authors.

## Critical analysis of key reviews in this area

Table 1 outlines the key reviews published to date that provide examples of the LLM applications in diagnostic medicine.

**Table 1:** Critical analysis of key reviews related to the LLM applications in diagnostic medicine

Authors	Study title	Area / Focus	Outcomes	Challenges
Eysenbach, Gunther [13]	The Role of Large Language Models in Diagnostic Medicine: A Literature Review with a Focus on Digital Pathology	To examine the current state of knowledge regarding the use of LLMs for diagnostic medicine, with a particular emphasis on their applications in digital pathology.	Studies have reported improved diagnostic accuracy and efficiency when pathologists incorporate LLM-based tools in their workflow.	LLMs heavily rely on the training data available to them, and biases in the data can result in biased or erroneous outputs. Efforts are required to ensure diverse and representative training datasets. - Limited interpretability of LLMs in understanding the underlying rationale for their predictions is a significant challenge, particularly in complex digital pathology cases. - Data privacy, security, and ethical concerns arise when integrating LLMs in clinical settings, emphasizing the need for robust frameworks and guidelines.
Muftić, Fatima et al. [9]	Title: Review of ChatGPT-based Diagnostic Medicine Applications	To explore the current state of knowledge regarding the use of ChatGPT in diagnostic medicine and its specific applications within this field	- ChatGPT can serve as a conversational agent, providing clinicians with real-time access to medical knowledge and literature, aiding in clinical decision-making	ChatGPT’s responses are generated based on statistical patterns in the training data and may lack contextual understanding or accuracy in specific medical scenarios.
Hariri, Walid [5]	Lack of Contextual Understanding in ChatGPT Responses	This study examined the contextual understanding of ChatGPT in the context of medical diagnoses.	It revealed limitations in the model’s ability to accurately interpret and respond to nuanced clinical scenarios, leading to potential inaccuracies or incomplete information.	The study emphasized the importance of cautious interpretation and validation of ChatGPT-generated responses by healthcare professionals.
Ma, Y [14].	The potential application of ChatGPT in gastrointestinal pathology	This study evaluated the biases present in ChatGPT responses by analyzing the model’s outputs in various medical scenarios.	The study highlights ability to summarize patients’ charts, its potential application in Digital Pathology, education, and research.	The study mentions the potential bias based on the datasets used in its training, the requirement of sufficient input information, as well as concerns related to bias, transparency, and generating inaccurate content.
Gregory Brennan [15]	Using ChatGPT to Write Pathology Results Letters	Utilizing ChatGPT to generate pathology results letters could automate the process, saving time and effort for pathologists.	ChatGPT lacks true understanding and context, which can be critical in pathology reports. Pathology findings may vary significantly depending on patient history, clinical context, and the specific case, and an AI language model may not be able to fully grasp these subtleties.	LLM models may struggle to handle rare or complex cases that require expert knowledge and interpretation. Uncommon findings might not be adequately covered in the training data, leading to potentially incorrect or inadequate results.
Sun et al. [2]	PathAsst: Redefining Pathology through Generative Foundation AI Assistant for Pathology	The researchers present PathAsst as a generative foundation AI assistant designed to improve diagnostic and predictive analytics in pathology.	PathAsst leverages the capabilities of the ChatGPT/GPT-4 language model, generating over 180,000 instruction-following samples. Additionally, they devise pathology-specific instruction-following data to allow PathAsst to interact effectively with pathology-specific models, enhancing its diagnostic capabilities.	The use of large language models and multimodal techniques can potentially enhance the accuracy and efficiency of pathology diagnostics, leading to improved patient care. However, to fully understand the findings and the impact of PathAsst, it is essential to read the full research paper, including the methodology, experimental results, and potential limitations.
Sorin et al. [16]	Large language model (ChatGPT) as a support tool for breast tumor board	The aim of this study is to evaluate ChatGPT as a support tool for breast tumor board decisions making. We inserted into ChatGPT-3.5 clinical information of ten consecutive patients presented in a breast tumor board in our institution. We asked the chatbot to recommend management.	ChatGPT’s recommendations were like the tumor board’s decisions. Mean scores while grading the chatbot’s summarization, recommendation, and explanation by the first reviewer were 3.7, 4.3, and 4.6 respectively. Mean values for the second reviewer were 4.3, 4.0, and 4.3, respectively.	Authors present initial results on the use of an LLM as a decision support tool in a breast tumor board. Given the significant advancements, it is warranted for clinicians to be familiar with the potential benefits and harms of the technology.

From the analysis, we found below key areas to form a generalized framework:

- Define Diagnostic Tasks: Clearly define the diagnostic tasks you want to evaluate using LLMs. These could include tasks such as medical image captioning, medical report generation, disease prediction from clinical notes, etc.
- Data Collection: Gather relevant datasets for the chosen diagnostic tasks. Ensure that the datasets are diverse, covering various medical conditions and modalities.
- Preprocessing: Preprocess the data to make it compatible with the LLM input format. For images, this might involve converting them into a format suitable for processing, and for text-based tasks, ensure the text data is appropriately cleaned and tokenized.
- Model Selection: Choose suitable LLM architectures or pre-trained models for your tasks. This might include models fine-tuned for medical applications or general-purpose LLMs adapted to the medical domain.
- Training and Evaluation: Train LLMs on the defined diagnostic tasks and evaluate their performance using relevant metrics. For image-related tasks, you may need to integrate the LLM with other models, such as convolutional neural networks (CNNs).
- Comparison with Baseline Models: Compare the performance of LLMs with baseline models or traditional approaches commonly used in diagnostic medicine. This provides context and helps identify the added value of LLMs.
- Transfer Learning and Fine-Tuning: Explore the use of transfer learning and fine-tuning on domain-specific medical data. Pre-trained LLMs might capture generic medical knowledge, but fine-tuning can enhance their performance on specific diagnostic tasks.
- Interpretability: Investigate the interpretability of LLMs in the medical context. Understand how decisions are made and explore methods to provide transparent insights to healthcare professionals.
- Clinical Validation: Collaborate with healthcare professionals to validate the clinical relevance and utility of LLM-based diagnostic tools. Understand their perspectives on integrating such tools into real-world medical practice.
- Ethical Considerations: Address ethical considerations related to patient data privacy, bias, and fairness in the deployment of LLMs in healthcare settings.

## Discussion

This review highlights the potential benefits of integrating LLMs in diagnostic medicine, such as improved clinical decision support, enhanced patient education, and disease surveillance capabilities. LLM can provide real-time access to medical knowledge, assist in telemedicine applications, and empower patients to actively participate in their healthcare. Furthermore, LLMs can contribute to disease surveillance efforts by monitoring various data sources for early detection of outbreaks and dissemination of public health information [5, 9, 13].

Image-based diagnostics and laboratory-based diagnostics are two fundamental approaches to clinical diagnosis, each with its own strengths and limitations. Understanding these differences is crucial for LLM applications to effectively support healthcare professionals in the diagnostic process.

Image-based diagnostics involve analysing medical images, such as X-rays, CT scans, MRIs, and ultrasound images, macroscopic skin lesions, dermoscopic, histopathological, and cytological images to identify abnormalities or patterns suggestive of disease. LLMs can play a significant role in image-based diagnostics by:

i. Image analysis and feature extraction: LLMs can be trained to automatically identify and extract relevant features from medical images, such as tumours, fractures, or specific anatomical landmarks. This can assist radiologists in detecting subtle abnormalities that might be missed by the human eye.
ii. Image classification and diagnosis: LLMs can be trained to classify medical images based on the presence or absence of specific diseases or conditions. This can provide rapid preliminary diagnoses and help prioritize cases for further investigation.
iii. Image segmentation and lesion characterization: LLMs can be used to segment medical images into different tissue types or to identify and characterize lesions. This can provide valuable information for treatment planning and prognosis.

Laboratory based diagnostics involve analysing blood, tissue, or other bodily fluids to measure the levels of specific biomarkers or to detect the presence of pathogens. LLMs can support lab-based diagnostics by:

i. Analysing laboratory test results: LLMs can be trained to interpret lab test results in the context of a patient’s medical history and other clinical data. This can help clinicians identify patterns and make accurate diagnoses.
ii. Predicting disease risk: LLMs can be used to develop predictive models that estimate a patient’s risk of developing certain diseases based on their genetic and lifestyle factors. This information can be used for preventive measures and early intervention.
iii. Identifying new biomarkers: LLMs can be used to analyse large datasets of lab test results to identify new biomarkers that may be associated with specific diseases. This can lead to the development of new diagnostic tests.

Table 2 provides a summary of these key differences between the Image-based and Laboratory-based diagnostics with an outline of the possible applications of LLM in both diagnostic approaches.

**Table 2:** Key differences between Image-based and Laboratory-based Diagnostics with a focus on LLM applications

Feature	Image-based diagnostics	Laboratory-based diagnostics
Data source	Medical images	Blood, tissue, other bodily fluids
Analysis method	Visual analysis, image processing, AI	Biochemical analysis, genetic testing, pathogen detection
Strengths	Non-invasive, rapid, can visualize anatomical structures	More specific, quantitative, can detect biochemical changes
Limitations	Subjective, requires trained specialists, prone to artifacts	Invasive, time-consuming, may not be specific to a single disease
LLM applications	Image analysis, classification, possibly segmentation and lesion characterisation	Analysing test results, predicting disease risk, identifying new biomarkers

Overall, both image-based and laboratory-based diagnostics play crucial roles in modern healthcare. LLMs have the potential to revolutionise both approaches by providing automated analysis, improved accuracy, and personalized insights. By integrating LLMs with existing diagnostic workflows, we can move towards a future of faster, more accurate, and more efficient medical diagnosis.

The are a number of LLM and large multimodal models (LMMs) available that are trained on majority of image-based and laboratory-based diagnostics which are reviewed by recent papers [17–19]. Some of these LLMs and LMMs are also trained on histopathology images such as Llava-med [20] and others [21–24]. These models work entirely differently to the classical machine learning (ML) and deep learning (DL) algorithms due to their pretraining and advance architecture enabling them conversational features to answer all possible questions one could ask about image-based or laboratory-based diagnostic scenarios.

However, this review also found several critical limitations of LLMs applications in diagnostic medicine. One key limitation is the reliance of LLMs on training data, which can introduce biases and inaccuracies. The lack of contextual understanding and interpretability of the model’s outputs in specific medical scenarios poses challenges. Ethical concerns related to patient privacy, data security, and the responsible use of AI in healthcare must also be thoroughly examined [10–12].

The integration of LLMs in diagnostic medicine has generated significant interest and excitement due to its potential to improve clinical decision-making and patient care. However, a critical examination of the applications of LLMs in diagnostic medicine reveals several important considerations that must be carefully evaluated [5, 9, 11–14].

### Limitations of contextual understanding

While LLMs can generate coherent and contextually relevant responses, it lacks true understanding of the underlying medical concepts. The model’s responses are based on statistical patterns learned from training data, which may not always capture the intricacies and complexities of medical diagnoses. In situations where deep contextual understanding is crucial, such as rare diseases or complex patient cases, relying solely on LLM’s responses may lead to inaccurate or incomplete information [14, 25–27].

### Interpretability

LLMs’ ‘s black-box nature lacks expalinability and presents challenges in understanding the rationale behind its generated responses. The model does not provide transparent reasoning for its decisions, making it difficult for healthcare professionals to trust and validate its recommendations. This lack of interpretability hampers the model’s utility in critical decision-making scenarios where clinicians require a clear understanding of the reasoning behind the suggested diagnoses or treatment options [28]. Advances in evolutionary and genetic algorithm, which allow the performance to be verified on several benchmarks offer possible solutions to this concern [29, 30].

### Reliance on training data

The performance of LLMs is heavily reliant on the quality, diversity, and representativeness of the training data. Biases and inaccuracies present in the data can be inadvertently learned and perpetuated by the model, leading to biased outputs and potential disparities in diagnoses. The responsibility lies with the developers and researchers to ensure that the training data is carefully curated, scrutinized for biases, and continuously updated to reflect the diversity of patient populations and clinical scenarios.

### Ethical considerations

The integration of LLMs in diagnostic medicine raises important ethical considerations. Privacy and security of patient data must be prioritized to protect sensitive health information. Proper consent mechanisms should be established to ensure patients are aware of their data being used by the model. Additionally, ethical concerns surrounding the potential liability and accountability of AI systems in healthcare need to be carefully addressed to avoid potential harm to patients and legal implications.

### Impact on healthcare professionals

The introduction of LLMs in diagnostic medicine may lead to concerns regarding the professional autonomy and decision-making abilities of healthcare professionals. There is a risk of over-reliance on the model’s suggestions, potentially diminishing critical thinking and independent clinical judgment. Care must be taken to ensure that LLMs serve as a valuable tool to augment the expertise of healthcare professionals rather than replacing their crucial role in the diagnostic process.

### Validation and real-world testing

While ChatGPT shows promise in various applications, there is a need for rigorous validation and real-world testing to assess its performance, reliability, and impact on patient outcomes. Controlled studies, comparative evaluations, and clinical trials are necessary to understand the true effectiveness of ChatGPT in improving diagnostic accuracy, reducing errors, and enhancing patient care.

## Conclusions

The use of LLMs in diagnostic medicine holds significant promise, offering potential benefits in clinical decision support, patient engagement, and public health efforts. However, critical considerations must be addressed to ensure the responsible and effective integration of LLMs. This review provides insights into the strengths, limitations, and ethical implications associated with the use of LLM in diagnostic medicine, ultimately highlighting the need for further research, validation, and collaboration between AI developers and healthcare professionals.

While LLM-based applications in diagnostic medicine offer potential benefits, a critical assessment reveals several limitations and challenges that must be addressed. The model’s lack of true contextual understanding, limited interpretability, and reliance on training data introduce uncertainties and potential inaccuracies in its responses. Biases in the data can lead to biased outputs, raising concerns about equitable and accurate diagnoses. Ethical considerations, including patient privacy and the responsible use of AI, require careful attention. The impact on healthcare professionals’ autonomy and the need for robust validation and real-world testing further highlight the complexity of integrating LLM in diagnostic medicine [28]. By addressing these concerns, LLM can be effectively integrated as a valuable tool to augment clinical decision-making, improve patient care, and advance the field of diagnostic medicine [2, 3, 5, 11, 15, 16].

To harness the potential of LLMs, a collaborative and iterative approach is necessary. Developers, healthcare professionals, and regulatory bodies must work together to refine the model, address its limitations, and establish transparent guidelines for its use. Further research and validation studies are needed to assess the model’s performance, reliability, and impact on patient outcomes. Ultimately, the responsible integration of LLM-based applications in diagnostic medicine can enhance clinical decision-making and patient care, but careful consideration of the critical factors is essential for its successful implementation [1–3, 5, 11, 15, 16].

## Future considerations

As the field of diagnostic medicine continues to evolve, the integration of LLMs and other large language models holds significant potential. To ensure the responsible and effective use of LLMs in diagnostic medicine, the following considerations should be addressed in future research and implementation:

1. Contextual Understanding and Interpretation: Efforts should be focused on enhancing LLM’s contextual understanding and improving its ability to provide transparent explanations for its generated responses. It is important that the data used to train LLM also include a large amount of medical data. Advancements in natural language processing and explainable AI techniques can contribute to better interpretability, enabling healthcare professionals to trust and validate the model’s recommendations [9, 25].
2. Collaborative Model Development: Collaboration between AI developers, healthcare professionals, and domain experts is crucial for the successful integration of LLMs in diagnostic medicine. It is very important for healthcare professionals be involved in the tuning of the datasets as well as in the validation mechanisms. Joint efforts can help refine the model’s training data, incorporate domain-specific knowledge, and ensure that the model aligns with the clinical workflow and requirements of healthcare settings [11, 26].
3. Bias Detection and Mitigation: To minimize biases in LLM’s outputs, ongoing efforts are needed to detect and mitigate biases in training data. Diverse and representative datasets should be used, and strategies should be implemented to identify and correct biases as they emerge. Regular audits and evaluation of the model’s performance with respect to fairness and equity are essential [31].
4. Integration with Clinical Decision Support Systems: LLMs can be integrated with existing clinical decision support systems to enhance their capabilities. By leveraging the model’s conversational abilities, it can provide real-time, evidence-based recommendations, assist in diagnostic reasoning, and offer educational resources to support healthcare professionals in making informed decisions [32].
5. Longitudinal Studies and Real-World Evaluation: Long-term studies and real-world evaluations are necessary to assess the impact of LLM-based applications on patient outcomes, healthcare costs, and clinical workflow optimization. These studies should involve diverse patient populations and consider different healthcare settings to validate the model’s effectiveness and generalizability [13, 25, 31, 32].
6. Regulatory and Ethical Guidelines: Clear guidelines and regulations are needed to govern the integration of LLMs in diagnostic medicine. Ethical considerations related to patient privacy, data security, consent mechanisms, and the responsible use of AI should be addressed. Regulatory bodies and professional societies should collaborate to establish standards and frameworks that ensure the ethical and safe implementation of LLM-based applications [3, 4, 32].
7. User Feedback and Iterative Improvement: Continuous user feedback and iterative improvement of LLMs are essential to refine the model’s performance and address its limitations. Healthcare professionals’ input should be actively sought to improve the accuracy, relevance, and usefulness of the model’s responses in real-world clinical scenarios [3–6].The intended benefits should be defined and evaluations to check their fulfilments are essential.

By considering these future considerations, LLM-based diagnostic medicine applications can evolve into valuable tools that augment clinical decision-making, improve patient outcomes, and advance the field of diagnostic medicine [1, 13, 25, 26].

## Data Availability

Not applicable.
